# Chemical Modification of Cellulose Fibers for Sustainable Food Packaging: Structure–Property–Sustainability Relationships

**DOI:** 10.3390/ma19061124

**Published:** 2026-03-13

**Authors:** Marcin H. Kudzin, Zdzisława Mrozińska, Jerzy J. Chruściel, Joanna Olczyk, Monika Sikora, Edyta Sulak, Anetta Walawska

**Affiliations:** Łukasiewicz Research Network—Lodz Institute of Technology, 19/27 Marii Skłodowskiej-Curie Street, 90-570 Lodz, Poland; zdzislawa.mrozinska@lit.lukasiewicz.gov.pl (Z.M.); monika.sikora@lit.lukasiewicz.gov.pl (M.S.); anetta.walawska@lit.lukasiewicz.gov.pl (A.W.)

**Keywords:** cellulose fibers, esterification, etherification, phosphorylation, oxidation, degree of substitution (DS), surface-enriched modification, structure–property relationships, food contact materials, recyclability, chemical safety

## Abstract

Cellulose fibers offer renewable sourcing and an established recycling infrastructure for food packaging applications. Their hydroxyl groups bind water strongly, which causes dimensional instability and compromises barrier performance at elevated humidity. Chemical modification targets this limitation through controlled changes to hydroxyl reactivity, surface charge, and interfiber hydrogen bonding. This review covers four principal covalent modification routes: esterification, etherification, phosphorylation, and oxidative functionalization. The spatial localization of functional groups—surface-enriched versus bulk modification—is treated as a cross-cutting analytical parameter governing the translation of molecular chemistry into barrier performance, mechanical behavior, and recyclability. We emphasize how molecular parameters (degree of substitution (DS), charge density, and the spatial distribution of functional groups) translate into barrier properties, mechanical performance, and grease resistance under realistic service conditions. Two practical constraints define the design space. Bulk modifications that penetrate the fiber wall can release reagents or by-products into food (non-intentionally added substances, NIASs), whereas surface-confined chemistry reduces this risk substantially. Modifications that resist repulping or introduce persistent contaminants damage recyclability. Life cycle impacts often derive more from processing steps (mechanical fibrillation, solvent use, and multi-stage washing) than from feedstock selection. We focus on three deployment-relevant outcomes: performance retention above 75% relative humidity, migration risk under food contact regulations, and compatibility with industrial fiber recycling. The aim is to identify strategies that can move from laboratory demonstration to production-scale implementation.

## 1. Introduction

Fiber-based packaging has expanded from a niche material to a widespread alternative in food applications. The European Single-Use Plastics Directive (Directive (EU) 2019/904) has established policy mechanisms favoring renewable and recyclable packaging [1]. Chemical safety concerns, particularly around food contact chemicals (FCCs) and their migration into food, have gained prominence in packaging design [2,3]. Waste reduction targets, chemical risk management, and circularity objectives have converged to push the industry toward simpler, recyclable packaging constructions.

Cellulose fibers come from renewable sources and benefit from established collection and recycling infrastructure. However, the same properties that enable strong fiber-to-fiber bonding (high water affinity and strong hydrogen bonding) create problems for moisture, grease, and vapor barriers [4,5]. Multilayer constructions and polymeric coatings can address these performance gaps but add chemical complexity, obscure material composition, and often damage recyclability [2,4].

Chemical modification offers an alternative approach by introducing functional groups directly into the fiber substrate through controlled substitution rather than adding separate barrier layers. The chemistry can be targeted to the surface or distributed through the bulk fiber structure. When appropriately designed, this approach improves barrier and mechanical performance while maintaining recyclability and chemical transparency [4,5,6].

Covalent and ionic modifications of cellulose fibers are analyzed here in terms of their effects on fiber network performance in food packaging. Degree of substitution (DS), functional group chemistry, and spatial distribution (surface vs. bulk) serve as the primary analytical parameters. These molecular variables shape barrier properties under variable humidity, mechanical behavior, aging characteristics, migration risks, and recyclability [2,4,6].

Existing reviews of cellulose modification tend to optimize either barrier performance [4], chemical safety [3], or sustainability in isolation [7]. No current review integrates all three evaluation layers—molecular chemistry and spatial distribution of functional groups, functional packaging performance under realistic humidity conditions, and food contact safety together with circularity constraints—within a single analytical framework applied to fiber-based systems [1,2,6].

This review addresses that gap. Its unique analytical perspective lies in treating degree of substitution (DS) and surface-versus-bulk localization not as descriptive parameters but as controllable design variables that simultaneously determine barrier performance, migration risk, and recyclability [2,3]. Rather than cataloguing modification chemistries, this review asks which molecular design choices enable deployment-relevant performance above 75% relative humidity while remaining compatible with food contact regulations and industrial fiber recycling? The answer requires integrating chemistry, safety, and circularity—which the existing literature treats separately [2,3].

### 1.1. Fiber-Based Food Packaging in the Context of Sustainability

Packaging policy now prioritizes packaging solutions that reduce persistent pollution, simplify material flows, and enable high-quality recycling. The EU Single-Use Plastics Directive (EU) 2019/904 has accelerated the adoption of alternative packaging in affected product categories [1]. Circularity policy has also intensified focus on chemical safety: recycling can concentrate hazardous constituents and elevate migration exposures, directly linking chemical risk management to circular economy objectives [2].

The PFAS case is instructive. Denmark has prohibited PFAS use in paper and board food contact materials (with limited exceptions for functional barriers), demonstrating how even high-performing chemicals can become regulatory liabilities when persistence and accumulation are factored in [7]. Broader PFAS restriction initiatives under REACH, along with parallel policy streams on hazardous chemicals in packaging, make it clear that barrier strategies relying on persistent fluorinated chemistries face an uncertain future [8].

Fiber-based solutions (paper, board, and molded fiber) are expanding across food packaging segments, but unmodified cellulose remains a limiting factor. Native fiber networks absorb moisture quickly, swell, and lose dimensional and mechanical stability under humid conditions; they also provide weak barriers against water vapor and grease without additional treatment [4,5]. These limitations explain why industrial packaging has historically depended on coating and layering strategies. There is now a policy preference for building functionality into the fiber itself, where technically feasible, rather than relying on external layers—and this is why the current policy progresses toward intrinsic, fiber-centric routes to functionality where feasible [2,4].

### 1.2. Why Chemical Modification of Cellulose Matters

Chemical modification works because the anhydro-glucose unit offers multiple reactive hydroxyl sites (C2, C3, and C6), and the degree of substitution (DS) serves as a quantitative parameter linking molecular structure to macroscopic performance [4]. Compared to blending additives or applying post-coatings, chemical modification can adjust polarity and hydration at the fiber surface, adjust surface charge and specific interactions with water/lipids, and alter fiber–fiber bonding and network consolidation during forming and drying. All of these changes influence barrier and mechanical properties [4,5,6].

Where and how uniformly functional groups are introduced matters. Surface-selective modification can deliver significant interfacial effects with minimal disruption of the bulk fiber structure, which matters when recyclability and repulpability are priorities [6,9]. Bulk modification, by contrast, may be needed for durable performance under repeated stress, but it carries higher risks for end-of-life compatibility, and chemical safety changes in extractables, the formation of non-intentionally added substances (NIASs), and effects on recycling behavior all become more likely [2,6]. We treat cellulose chemistry as a controllable design parameter rather than an inherent sustainability limitation [2,6].

## 2. Cellulose Fibers for Food Packaging: Structure and Constraints

### 2.1. Hierarchical Structure of Cellulose Fibers

Cellulose fibers used in paper/board and molded-fiber packaging are hierarchical, water-structured materials rather than “solid filaments”: the macroscopic fiber (typ. millimeter scale) is built from a multilayer cell wall containing oriented microfibrils, which themselves are aggregates of elementary fibrils composed of cellulose I crystallites and amorphous domains. This hierarchy creates a coupled structure–transport problem: voids and capillaries dominate gas/liquid transport, while hydrogen-bonded microfibrils dominate stiffness and network cohesion [10,11].

At the molecular level, each anhydro-glucose unit (AGU) presents three hydroxyl groups (C2-OH and C3-OH as secondary alcohols; C6-OH as a primary alcohol), plus a reducing end that can be exploited for specific derivatizations. Reactivity is not uniform: under many heterogeneous conditions relevant to fibers and fibrils, apparent reactivity trends are commonly reported as C6-OH > C2-OH > C3-OH, with C3 often hindered by steric and intrachain hydrogen bonding [12,13]. This “reactivity anisotropy” is one reason why low-DS surface reactions can still meaningfully change wettability and interfiber interactions without fully transforming the bulk fiber wall [10,14,15].

Hydrogen-bond topology matters because it explains why cellulose is simultaneously strong and moisture-sensitive. The dense H-bond network stabilizes cellulose I packing, yet water can compete for accessible -OH sites in amorphous and surface regions, driving fiber wall swelling and plasticization. In practical terms, the same -OH chemistry that enables interfiber bonding and dry strength also creates the primary constraint for wet stability and water vapor barrier performance [11,16].

### 2.2. Chemical and Physical Limitations in FCM Applications

Unmodified cellulose packaging equilibrates at approximately 8 wt.% water at 23 °C/50% RH, which is sufficient to measurably shift stiffness, strength, and dimensional stability during conversion and use [17,18]. Barrier limitations follow directly from porosity and moisture-coupled transport. For the oxygen barrier, porous fiber networks generally show a very high OTR relative to dense polymer films. A widely used performance benchmark for many food packaging cases is an OTR < 10 cm^3^·m^−2^·day^−1^·bar^−1^ at 23 °C/50% RH, a target that uncoated paper often cannot reach because gas transport is dominated by interconnected pores rather than diffusion through a dense phase [19,20,21]. For water vapor, uncoated paper/board typically exhibits a high WVTR, and the representative literature examples show WVTR values on the order of ~10^3^ g·m^−2^·day^−1^ (condition-dependent), i.e., far above what is needed for moisture-sensitive foods—again reflecting transport through porous, hygroscopic structures [20,22].

Grease and oil resistance are a distinct constraint because fats can penetrate porous networks even when the oxygen barrier is improved. Grease resistance is often assessed using KIT values (1–12), where higher values indicate better resistance; uncoated paper can perform poorly (near “no resistance”), while many functional systems have historically relied on fluorinated chemistries to reach high KIT ratings—precisely the class now under strong substitution pressure [23,24]. Cellulose packaging performance cannot be reduced to hydrophobicity alone: grease resistance, water resistance, and vapor resistance are related but not equivalent functions, and each has different implications for recycling and chemical safety [23,25].

Hornification—the irreversible loss of swelling capacity after drying—reduces bonding potential upon rewetting and shifts fiber responses to chemical treatments. This is directly relevant to FCM performance and recyclability in systems subjected to thermoforming or repeated humidity cycling [26,27].

### 2.3. Native vs. Modified Cellulose: Design Space

The “design space” for fiber-based FCM can be understood as a set of chemically addressable levers that tune (1) surface energy and hydration, (2) charge and specific interactions, (3) interfiber bonding density, and (4) pore structure evolution during forming/drying. Chemical modification is powerful because it can act on several levers at once, but it must be engineered with spatial precision: a small change at the surface can shift wetting and capillary flow, while a bulk change can shift fiber wall swelling and mechanical response [10,14,27].

A practical way to formalize this design space is to separate surface-selective vs bulk modification. Surface-selective reactions exploit the fact that, under heterogeneous conditions, only a fraction of -OH sites are accessible, often leading to lower effective DS thresholds for measurable property shifts (e.g., altered wettability, reduced water uptake, and modified interfiber friction) without fundamentally changing the fiber interior. This is attractive for packaging because it can preserve repulpability and recycling behavior while still improving functional performance [10,14]. By contrast, bulk modification can deliver more persistent performance under humidity cycling but risks altering fiber flexibility, bonding, and disintegration behavior, which can translate into end-of-life penalties unless the chemistry is deliberately designed for reversibility or recyclability [20,26].

Not all “modification” must be covalent. Ionic complexation (e.g., with multivalent cations) and polyelectrolyte interactions can tune surface charge and hydration layers, but these interactions are often humidity-sensitive and may relax over time in contact with food simulants—creating a need to link the chemistry to aging and migration-relevant stability rather than reporting only initial barrier values [10,25,27].

## 3. Chemical Modification Strategies for Cellulose Fibers

Cellulose fibers can be modified through covalent and ionic reactions that change surface accessibility, polarity, and interaction behavior [28]. In food packaging, modification strategies must deliver functional performance while controlling the degree of substitution (DS), localizing reactions spatially, maintaining stability under use conditions, and meeting recycling and food contact safety requirements [2,29,30]. Packaging modifications usually work at a DS below 0.3. Small chemical changes in this range can shift wettability, interfiber interactions, and moisture response without disrupting fiber morphology or pulping behavior [30,31]. This section focuses on the modification methods that have been most thoroughly studied in fiber-based systems and for which structure–property relationships can be meaningfully discussed.

This section covers four principal covalent modification routes—esterification and acylation (Section 3.1), etherification and cationic/anionic functionalization (Section 3.2), phosphorylation including ionic complexation (Section 3.3), and oxidative functionalization (Section 3.4)—with TEMPO-mediated oxidation as the primary documented example. The spatial localization of functional groups—surface-enriched versus bulk modification—is treated as a cross-cutting analytical parameter within each route (Section 3.5). Structure–property relationships in modified cellulose fibers, including the influence of chemical structure on fiber–fiber interactions, are addressed in Section 4.

**Figure 1:**
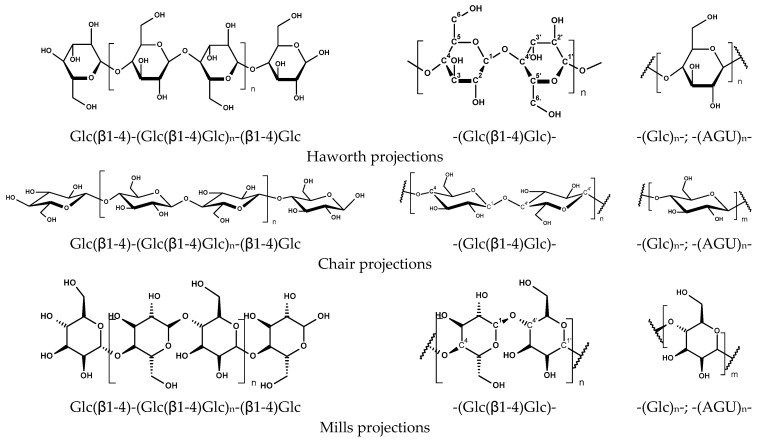
Cellulose structure presented in Haworth, chair, and Mills projections [34,35,36]. Cellulose presents a reactive polysaccharide platform, illustrating the anhydro-glucose unit (AGU) and the primary hydroxyl positions (C2, C3, and C6) involved in common chemical modification strategies. Typical reactions discussed in this review—esterification, etherification, and phosphorylation—differ in regioselectivity and spatial localization within heterogeneous fiber systems, highlighting the distinction between average degree of substitution (DS) and surface-enriched modification. TEMPO-mediated oxidation is shown as an illustrative example of surface-selective oxidative functionalization [10,32,33].

At the molecular level, cellulose is a polysaccharide with hydroxyl groups at the C2, C3, and C6 positions that serve as reactive sites. The average degree of substitution (DS) is commonly reported, but in heterogeneous fiber systems, the spatial localization of reactions—particularly surface-enriched versus bulk modification—plays an equally important role in determining material performance. Policy-driven waste reduction, tighter chemical safety standards, and circularity targets that favor simpler, recyclable architectures are reshaping the field. Figure 1 provides a schematic overview of the key reactive positions in cellulose and the principal modification routes discussed in the following sections [10,32,33].

### 3.1. Esterification and Acylation of Cellulose Fibers

Esterification has been extensively studied for cellulose modification. It is synthetically accessible, and ester groups strongly affect hydrophilicity and surface energy. Acetylation is the classic example. Longer-chain acylations like fatty acid esters are now being explored for barrier enhancement in fiber packaging [37,38].

**Figure 2:**
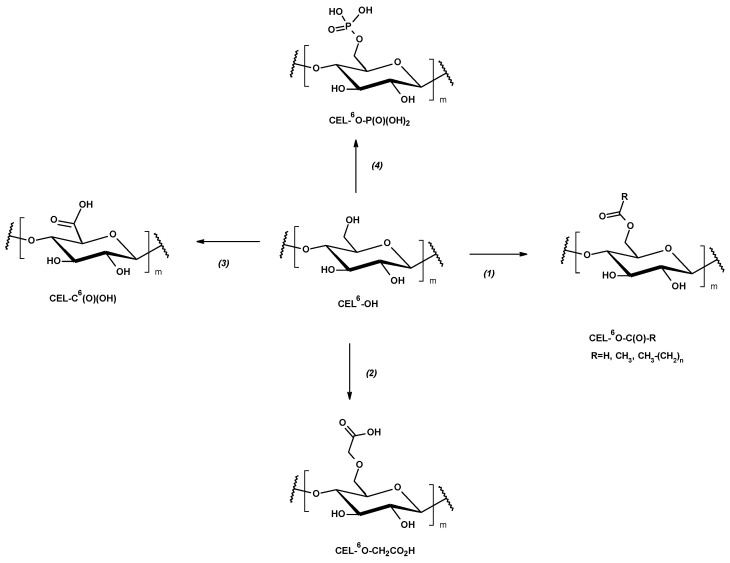
Minimal functional motifs representing common low-degree-of-substitution (low-DS) chemical modification strategies applied to cellulose fibers in packaging-relevant systems: (1) acetylation; (2) long-chain fatty-acid esterification; (3) etherification (CMC-type); (4) TEMPO-mediated oxidation and phosphorylation. The shown structures highlight the dominant functional groups introduced by each modification route without implying uniform bulk substitution [10,29,32,33,39].

To facilitate comparison between different modification routes discussed in the low-DS regime, Figure 2 summarizes the minimal functional motifs introduced into cellulose fibers by the most commonly applied esterification, etherification, oxidation, and phosphorylation strategies. These simplified structures are used throughout this section as chemical reference points for discussing structure–property relationships in fiber-based packaging systems [10,29,32,33,39].

Acetylation replaces hydrophilic hydroxyl groups with less polar acetyl groups, reducing water sorption and swelling. Acetylated cellulose fibers for paper and packaging typically show DS values from 0.05 to 0.3, well below fully substituted cellulose acetate grades used for plastics [37,40]. Even within this low-DS window, significant reductions in equilibrium moisture uptake and water vapor transmission have been observed, highlighting the non-linear relationship between surface chemistry and macroscopic performance [41].

Long-chain acylation (e.g., C8-C18 fatty acid esters) further amplifies hydrophobicity and can substantially improve grease resistance. Studies on fatty-acid-modified fibers report increases in KIT grease resistance values from near-zero for unmodified paper to KIT ≥ 7–10, depending on DS and fiber coverage [38,42]. However, such gains are often accompanied by reduced fiber–fiber hydrogen bonding, which can negatively impact dry strength and recyclability if bulk modification occurs [30,41].

For food contact safety, esterification is a chemically stable modification, provided that residual reagents and low-molecular-weight esters are effectively removed. Nevertheless, migration behavior depends strongly on DS and molecular weight of substituents: low-molecular-weight acetates can increase extractables if reaction control is poor, whereas higher acyl chains tend to be less mobile but may raise biodegradability and recycling concerns [2,43].

### 3.2. Etherification and Cationic/Anionic Functionalization

Etherification provides another modification route. Ether linkages stay intact across a wide pH and temperature range, which matters for applications with humidity cycling and thermal processing. Common etherified celluloses for fiber packaging include carboxymethyl cellulose (CMC), hydroxyethyl cellulose (HEC), and quaternary ammonium-functionalized celluloses [37,44].

CMC is particularly relevant because it introduces anionic carboxylate groups, increasing fiber surface charge and swelling capacity. Reported DS values for CMC used in papermaking contexts are typically DS ≈ 0.05–0.2, sufficient to enhance interfiber bonding and formation while maintaining fiber integrity [44]. However, increased hydrophilicity can be detrimental to water barrier performance, illustrating a key trade-off between mechanical reinforcement and moisture resistance [45].

Cationic etherification (e.g., quaternary ammonium groups) enables control over fiber surface charge and interactions with anionic species, including fats, proteins, and other food components. Low-DS cationic celluloses have been shown to improve retention and consolidation during forming, but their relevance for direct food contact is constrained by potential migration of low-molecular-weight cationic species and the need for toxicological clearance of quaternary ammonium groups [2,46].

In packaging-relevant systems, etherification is, therefore, often used indirectly, for example, as a surface treatment or additive to tune interfiber interactions rather than as a primary barrier-forming chemistry. The stability of ether linkages supports recyclability, but the increased water affinity associated with many etherified groups necessitates careful balancing when barrier performance is a primary design target [37,45].

### 3.3. Phosphorylation and Ionic Complexation

Phosphorylation introduces phosphate groups that change surface charge, hydration, and ion-binding capacity. Unlike esterification with neutral acyl groups, phosphorylation creates strongly anionic sites that form ionic complexes with multivalent cations like Ca^2+^, Mg^2+^, or Fe^3+^, enabling electrostatic structure control in fiber networks [32,47,48].

**Figure 3:**
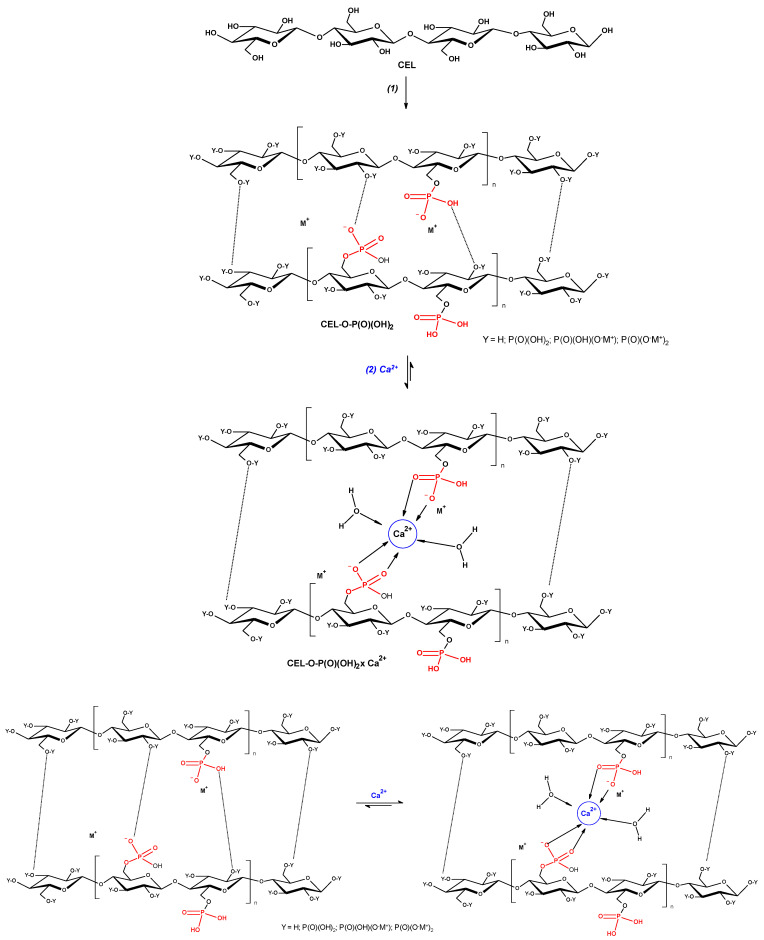
Phosphorylation and ionic crosslinking of cellulose fibers relevant to wet integrity and stability in fiber-based packaging systems: (1) formation of phosphate half-esters (P(V)) on cellulose hydroxyl groups and (2) multivalent-ion-mediated ionic bridging between anionic phosphate sites (illustrated for Ca^2+^), resulting in reversible network-level crosslinks [39,49]. Dotted lines present hydrogen bonds, and arrows symbolize coordination bonds. Calcium’s coordination number varies widely from 6 to 12, commonly ranging from 6 to 9 [50].

Phosphorylation introduces anionic functional groups onto cellulose fibers, enabling ion-mediated interactions that differ fundamentally from covalent crosslinking strategies. In fiber-based systems, such phosphate functionalities can participate in reversible ionic bridging with multivalent cations, providing a mechanistic basis for improved wet integrity and moisture tolerance, as schematically illustrated in Figure 3 [39,49].

Two broad classes of phosphate chemistry are reported in the literature: phosphate esters derived from pentavalent phosphorus (P(V)), which are generally stable under neutral and mildly acidic conditions, and phosphite- or phosphonate-related chemistries based on trivalent phosphorus (P(III)), which are more reactive but may raise additional concerns related to chemical stability and food contact safety [39,47].

Vapor-phase phosphorylation approaches provide an instructive illustration of how regioselective cellulose modification can be achieved under diffusion-limited conditions. Kudzin et al. demonstrated that PCl_3_ vapor treatment enables highly selective modification at the primary 6-hydroxyl position, leading to the formation of cellulose-6-phosphate (3) species that can subsequently undergo ionic complexation with Cu^2+^ ions, without full fiber dissolution or extensive bulk substitution [51]. This example highlights the role of reaction environment and accessibility in decoupling surface functionalization from fiber wall chemistry.

Despite such advances, packaging-relevant research overwhelmingly focuses on P(V)-based phosphate systems, which offer a more favorable balance between chemical stability and functional performance. Reported degrees of substitution are typically below DS ≈ 0.1, a range sufficient to generate measurable surface charge densities while avoiding excessive fiber swelling and loss of mechanical integrity [48].

Ionic complexation of phosphorylated cellulose with divalent cations can further reduce water uptake and increase network cohesion by introducing reversible ionic crosslinks. For instance, Ca^2+^-complexed phosphate celluloses have been shown to exhibit reduced swelling and improved wet strength relative to unmodified fibers, while largely retaining repulpability due to the non-covalent nature of the interactions [32,49]. Such behavior is particularly attractive for molded fiber packaging, where dimensional stability under humid or intermittently wet conditions is critical.

Phosphorylation occupies an intermediate position with respect to sustainability and food contact performance requirements. It avoids persistent fluorinated chemistries and can remain compatible with fiber recycling streams, yet it introduces inorganic phosphorus species that may accumulate during repeated recycling cycles. Careful control of phosphorus content, binding strength, and potential leachability is required, and long-term data describing behavior in closed recycling loops remain limited [2,3].

### 3.4. Oxidative Functionalization

TEMPO (2,2,6,6-tetramethylpiperidine-1-oxyl)-mediated oxidation selectively converts C6 primary hydroxyl groups into carboxylate groups, yielding surface-charged cellulose nanofibrils (TOCNFs) at DS values typically in the range 0.05–0.15 [50]. This surface-selective reaction is the most extensively documented oxidative route in the packaging-relevant literature [33]. The resulting high surface charge drives fiber individualization and network densification upon drying, producing films and coatings with strong oxygen barrier performance under dry conditions [49,51]. Other oxidative approaches—including ammonium persulfate, periodate, and hypochlorite oxidation—exist but are less commonly applied in fiber-based packaging contexts [33]. In this review, TEMPO-mediated oxidation is discussed primarily as a reference system for surface charge effects on fiber–fiber interactions (Section 4.1) and as a performance benchmark for barrier properties (Section 4.2).

### 3.5. Degree of Substitution and Chemical Heterogeneity as Design Parameters

Degree of substitution (DS) provides a quantitative link between molecular-level chemistry and macroscopic performance, safety, and circularity outcomes. For fiber-based food contact materials, DS represents a spatially heterogeneous parameter that reflects reaction localization, accessibility, and fiber wall architecture rather than a simple stoichiometric average [10,30,47].

Heterogeneous cellulose systems exhibit surface-enriched substitution that dominates functional behavior even at low average DS. Studies demonstrate that DS values below 0.05–0.1 induce measurable changes in wettability, surface charge density, fiber–fiber friction, and water uptake when functional groups concentrate at accessible surface or near-surface regions [33,39,41]. Bulk DS values alone cannot predict performance; spatial distribution of substituents determines functional and environmental outcomes.

Chemical heterogeneity arises from three coupled factors: intrinsic reactivity differences between C2, C3, and C6 hydroxyl groups; diffusion limitations imposed by fiber wall ultrastructure; and reaction environment characteristics (heterogeneous slurry, vapor phase, or partially swollen systems) [32,47]. Materials with identical nominal DS may exhibit fundamentally different behavior in hydration, mechanical integrity, and aging stability when spatial distribution differs. This distinction matters for packaging applications where surface-dominated interactions control barrier properties and migration behavior, while bulk chemistry governs strength development and recyclability [2,10].

Surface-sensitive analytical methods—XPS, ToF-SIMS, and selective titration—consistently reveal functional group enrichment at fiber surfaces following heterogeneous modification, confirming that surface chemistry rather than bulk substitution dictates functional outcomes in fiber networks [35,48]. Low-DS, surface-selective modification strategies enhance performance while minimizing impacts on recycling and chemical safety.

At higher substitution levels (typically DS ≥ 0.2–0.3, depending on chemistry), increased substitution density disrupts inter- and intrafibrillar hydrogen bonding, reduces fiber conformability, and alters hornification behavior. These changes negatively affect dry strength, repulpability, and fiber yield in recycling loops [27,52]. Higher DS values also correlate with elevated risks of extractables, migration, and NIAS formation, particularly with low-molecular-weight reagents or labile linkages [2,3]. Maximization of DS rarely serves as a viable design objective for sustainable fiber-based FCM.

Chemical heterogeneity determines aging and durability. Modifications localized at highly accessible regions exhibit greater susceptibility to hydrolysis, ion exchange, or displacement under humid or food contact conditions, while deeply embedded substitutions persist longer but impact recyclability more strongly [49,53]. The optimal design window for many packaging applications lies in an intermediate regime: a DS sufficiently high to stabilize surface functionality during use yet low enough to preserve fiber wall integrity and end-of-life compatibility.

DS and chemical heterogeneity function as deliberate design parameters rather than secondary descriptors. Reporting practices should combine nominal DS values with information on reaction conditions, localization (surface versus bulk), and aging behavior to enable meaningful comparison across studies and to translate laboratory-scale modifications into industrially relevant, sustainability-by-design packaging solutions [10,30,47].

Chemical modification strategies for cellulose fibers—often described using qualitative labels such as “acetylated”, “oxidized” or “phosphorylated”—depend strongly on modification intensity and spatial distribution within the fiber wall for functional relevance in packaging applications. In heterogeneous fiber systems, relatively low degrees of substitution induce pronounced changes in wetting behavior, interfiber interactions, and transport properties when modification is surface-enriched [10,32,41].

To place the most widely used modification chemistries into a comparable design context, Table 1 summarizes representative ranges of degree of substitution reported for fiber-based systems, together with their dominant functional effects and primary packaging relevance. The reported values should be interpreted as practical literature windows rather than strict thresholds, as actual performance remains highly dependent on substrate type, processing history, humidity, and conditioning history [2,41,52]. Policy-driven waste reduction, tighter chemical safety standards, and circularity targets that favor simpler, recyclable architectures are reshaping the field.

## 4. Structure–Property Relationships in Modified Cellulose Fibers

### 4.1. Effect of Chemical Structure on Fiber–Fiber Interactions

Functional properties of fiber-based packaging—strength, barrier continuity, and dimensional stability—depend on fiber interactions within the wet web and during drying. Hydrogen bonding remains the dominant intermolecular binding mechanism, but macroscopic performance is governed by contact area, fiber conformability, surface chemistry, and the presence of polyelectrolytes or nanofibrillar binders. Chemical modification influences performance through changes in intrinsic surface energy and through modification of the bonding landscape, including the number and quality of fiber–fiber contacts [61,62]. The structure–property relationship is controlled by competing effects: hydration and swelling promote conformability and contact development. However, excessive water affinity weakens wet integrity and accelerates moisture-driven property drift. This balance is particularly evident in oxidized and anionic celluloses such as TEMPO-oxidized CNF, where carboxylate formation increases electrostatic repulsion in suspension but enhances network densification upon drying through increased fibril-level contact areas and stronger capillary consolidation [33]. Surface charge and counter-ion identity function as chemical process variables that modulate aggregation, drainage, and the evolution of interfiber contacts under papermaking conditions [47].

Surface-selective modification produces large effects relative to bulk substitution. Surface-sensitive techniques such as X-ray photoelectron spectroscopy demonstrate that heterogeneous reactions preferentially enrich fiber surfaces with functional groups. Even at low average degrees of substitution, these surface modifications can substantially alter interfiber friction, bonding kinetics, and wet-web rheology [38]. Tarrés et al. demonstrated that combining enzymatic CNF in bulk with TEMPO-oxidized CNF as a surface coating produces a hierarchical bonding architecture that delivers strength gains exceeding those achieved with either component alone. The result demonstrates the value of hierarchical chemical structuring through macro-fiber and nano-binder combinations rather than an increasing degree of substitution [63]. It should be noted, however, that TEMPO-mediated oxidation is only one of several approaches and that other oxidation methods may also be relevant. In addition to the popular TEMPO-mediated oxidation, which selectively converts C6 hydroxyl groups to carboxyl groups, there are a number of other methods that allow for the modification of cellulose for specific industrial and medical applications, including the ammonium persulfate (APS) method, periodic acid oxidation, nitrogen dioxide oxidation, hydrogen peroxide oxidation, and sodium hypochlorite oxidation. This review, however, does not focus on a detailed description of these methods. The authors would like to emphasize that TEMPO oxidation is merely a cited example for the purposes of the phenomenon described here [63].

### 4.2. Barrier Properties (Water, Grease, Vapor)

Barrier performance in fiber packaging is fundamentally limited by the porous, hydrophilic, and moisture-responsive nature of cellulose networks. Chemical modification influences barrier properties through two primary pathways: (1) altering pore topology and tortuosity via fiber–fiber consolidation and film or coating continuity and (2) modifying sorption–diffusion thermodynamics through changes in surface polarity and specific interactions with permeants such as water vapor, oxygen, and grease components [61].

Barrier performance in fiber-based packaging depends on both chemical functionality and moisture–structure interactions that control the integrity of the fiber or coating network [61,64]. Reported oxygen and water vapor transmission values should be interpreted in relation to relative humidity, coating continuity, and fiber consolidation, rather than as intrinsic material constants [22,61].

Table 2 presents the barrier properties of modified cellulose systems under conditions relevant to packaging. Representative barrier ranges are shown for chemically modified cellulose-based systems compared to nanocellulose systems. Nanocellulose systems differ fundamentally from natural cellulose fibers primarily in terms of structure, properties, and performance, exhibiting novel behaviors. Therefore, nanocellulose is not a direct extension of chemically modified fibers. For the oxygen barrier, nanocellulose-based layers (CNFs/CNCs/TOCNFs) can achieve extremely low oxygen permeability under dry conditions, but performance is typically humidity-sensitive due to plasticization and swelling. A representative dataset is reported for nanocellulose coatings applied by spin- and dip-coating: oxygen permeability values are in the range 0.12–24 mL·µm/(m^2^·24 h·kPa) at 23% RH, whereas at 50%, RH oxygen permeability can become too high to quantify for many thin coatings (with thicker dip-coated layers being less affected) [59]. This quantifies a key design rule: oxygen barrier gains from cellulose-based coatings are real, but stability above ~50% RH usually requires additional chemistry or multilayer shielding [65].

Moisture barrier (WVTR) is generally more challenging than the oxygen barrier for cellulose-derived layers. A consistent conclusion across reviews is that improvements in water vapor resistance typically require either (1) incorporation of hydrophobic phases or (2) surface chemistries that reduce water uptake without disrupting film cohesion. Wang et al. synthesized extensive data across CNM films and coatings and emphasized that barrier metrics must be interpreted alongside humidity conditions, thickness, and measurement standards—because apparent “best-in-class” values often collapse when RH increases [61].

Multilayer shielding approaches can further mitigate the humidity sensitivity of cellulose-based barriers; however, such constructions fall outside the substrate-level chemical modification scope of this review and are noted only for contextual comparison [66].

Beyond nanocellulose coatings, chemically modified micro-fibrillated cellulose systems also show how surface chemistry can increase barrier performance by reducing sorption and densifying the structure. Rodionova et al. reported that the surface chemical modification of micro-fibrillated cellulose improves barrier properties for packaging applications, with results that are strongly dependent on reaction severity and substitution distribution (surface vs bulk). This underscores that the way substitution is achieved can matter more than nominal DS alone [41].

### 4.3. Mechanical Performance of Modified Fiber Networks

Mechanical performance is controlled by the interplay between fiber intrinsic strength, network formation, bonded area, and stress transfer across the fiber–fiber interface. Among the four modification routes discussed in this review, esterification and acylation affect mechanical performance primarily through changes in interfiber hydrophobicity and reduced hydrogen bonding capacity [30,41,54]; etherification (particularly CMC-type) increases fiber swelling and bonded area under wet conditions [44,45]; phosphorylation contributes to wet integrity through ionic crosslinking with multivalent cations [32,49]; and oxidative functionalization (TOCNF) improves dry-state network densification and the bonded contact area through electrostatic-driven consolidation [33,63]. Chemical modification shifts this balance by changing fiber conformability and collapsibility during drying (1), interfacial adhesion (2), and (3) defect sensitivity through nano-scale reinforcement and surface bonding [62].

In fiber network engineering, strength improvements are most effectively achieved by increasing the effective bonded area. Maximizing chemical substitution is less effective. Nanofibrillar binders (particularly TOCNF) can act as a “mortar” that bridges microfiber contacts and reduces stress concentration at junctions. In the combined additive/coating strategy reported by Tarrés et al., paper strength increased markedly when enzymatic CNF was used in bulk and TOCNFs were applied as a coating, illustrating that hierarchical placement of chemically distinct cellulose fractions can deliver mechanical gains at lower chemical intensity than bulk substitution alone [63].

However, chemical modification can also reduce strength if it disrupts hydrogen bonding or introduces excessive hydrophobicity at fiber contact points. This trade-off is most acute for long-chain esterification/acylation strategies that increase grease resistance but may reduce interfiber cohesion [38,41]. Etherification (CMC-type) generally preserves or improves bonding through increased swelling but at the cost of reduced moisture resistance [44,45]. Phosphorylation at low DS maintains fiber morphology and can improve wet strength through ionic crosslinking [32,49], while oxidative functionalization (TOCNFs) improves network consolidation in dry conditions but shows limited wet mechanical stability without additional crosslinking [33,63]. The solution is surface-selective, patchy, or paired modification with an internal bonding strategy (e.g., CNF addition). Structure–property optimization typically requires controlling the spatial distribution of substituents, counter-ion chemistry, and the fraction of nanofibrillar binder rather than focusing only on DS as a scalar [33,41].

### 4.4. Aging, Stability, and Process Robustness

Aging in modified fiber packaging is dominated by moisture cycling, thermally activated relaxation, and structural “locking” processes such as hornification. Cellulose is moisture-sensitive. Even small chemical changes at the surface can cause noticeable shifts in strength and barrier performance over time—especially when humidity levels keep changing during storage and use [61,65].

The scientific literature indicates that modified cellulose (e.g., esters or ethers) exhibits a specific sensitivity to environmental factors, leading to a decrease in molecular weight, loss of functional groups, and deterioration of mechanical properties. Studies on cellulose acetate (CA) demonstrate that cyclic changes in humidity lead to deacetylation. The released acetic acid acts as a catalyst (autocatalysis), accelerating the hydrolysis of glycosidic bonds. This phenomenon results in a decrease in the degree of substitution (DS) and brittleness of the fibers. In the conservation literature, this phenomenon is referred to as “acetic acid syndrome” [61,65]. Research on carboxymethylcellulose (CMC) focuses on the stability of dispersions and powders. Prolonged exposure to oxygen and light (UV) leads to the formation of carbonyl and carboxyl groups, which alter the original modification profile. Low-DS modifications are susceptible to enzymatic attack by bacteria and fungi, resulting in a complete loss of solution viscosity during storage. These observations highlight that oxygen barrier claims must be validated after realistic conditioning histories, not only at a single RH/time point [60,65].

Ionic complexation provides one route to enhance wet integrity and reduce humidity-driven collapse by introducing reversible ionic crosslinks that stabilize fibrillar contacts. Benselfelt et al. showed that multivalent ions can markedly improve the wet integrity of cellulose nanofibril films. This offers a mechanistic basis for using controlled counter-ion chemistry (e.g., Ca^2+^/Mg^2+^/Al^3+^ in selected contexts) as a stability lever—provided that food contact safety and recycling compatibility are not compromised [49].

Hornification remains a critical factor influencing process robustness, particularly in recycled fiber streams and in networks subjected to repeated drying and wetting cycles. Hornification is a kinetically trapped state that reduces swelling capacity and changes fiber-water interactions, directly impacting formation, bonding, and barrier continuity in subsequent processing cycles. This reinforces the need to evaluate modified fibers under recycling-relevant conditioning and repulping scenarios [27].

Chemical modification is a powerful lever for tuning fiber–fiber interactions, barrier performance, and aging stability in cellulose-based packaging systems. However, the same molecular features that enable these functional gains—altered polarity, charge density, and interfacial chemistry—also define the potential for chemical migration, the formation of non-intentionally added substances (NIASs), and changes in bioavailability under food-contact-relevant conditions. Structure–property optimization cannot be decoupled from safety considerations, particularly for fiber-based materials intended for direct or prolonged food contact [2,3].

Regulatory frameworks increasingly emphasize chemical transparency and risk-based assessment of food contact materials. Chemical modification strategies must, therefore, be evaluated not only in terms of performance and sustainability but also with respect to migration behavior, chemical stability, and toxicological relevance throughout the use phase and recycling loops [3,67].

## 5. Chemical Modification and Food Contact Safety

### 5.1. Migration and NIAS Formation

Migration testing determines food contact safety for modified cellulose materials. What migrates into food under realistic use conditions (temperature, time, food type, and repeated use) and how this migration profile evolves during aging and recycling are the key questions. In EU practice, migration compliance is typically framed by the overall migration limit (OML), expressed as 10 mg/dm^2^ of contact surface (equivalent to 60 mg/kg food under the conventional 6 dm^2^/kg surface-to-mass assumption), alongside substance-specific migration limits (SMLs) where applicable [68,69].

For fiber-based packaging, the chemical inventory that can contribute to migration extends well beyond intentionally added reagents used for cellulose modification. It includes impurities, reaction side-products, hydrolysis products, oxidation fragments, residual catalysts, and printing-ink related compounds (when present), as well as processing aids and recycled-fiber contaminants. NIAS formation matters for cellulose chemistry because many reactions used to tune barrier and wet strength (e.g., esterification, cationization, phosphorylation, and oxidation) can generate low-molar-mass species and labile linkages that evolve under humidity/heat, yielding time-dependent migration profiles [3,70].

Migration testing is constrained by method uncertainty and worst-case conventions. The JRC guidance on migration test conditions emphasizes that analytical error in OML determination may be on the order of 2 mg/dm^2^ (12 mg/kg) for aqueous simulants and 3 mg/dm^2^ (20 mg/kg) for fatty simulants, highlighting why borderline OML results require careful interpretation and method control [68]. This is directly relevant to chemically modified cellulose, where low but non-zero extractables can cluster near the OML threshold depending on coating add-on, degree of substitution, and surface-to-volume ratio of the final article [71].

The NIAS-focused literature shows that unknowns are common in packaging systems—they are structurally expected in packaging systems. The widely cited review by Nerin et al. frames NIASs as arising from reaction/degradation processes and impurities, and it highlights the practical difficulty of applying a single universal workflow to identify unknown migrants due to the diversity of chemical classes and matrices. For modified cellulose, this is compounded by the porous nature of fiber networks (sorption/retention) and the possibility of migration from both fiber and coating layers under cyclic humidity [70].

For paper and board materials specifically, additional safety attention is driven by the history of recycled-fiber contaminants, particularly mineral oil hydrocarbons (MOHs) originating from printing inks and recycled paper. In real-market case monitoring, Lorenzini et al. reported that for egg pasta directly packed in recycled paperboard, MOSH migration reached ~30 mg/kg over 8 months at 20 °C, while accelerated storage at 40 °C produced comparable migration within ~1 week, illustrating strong temperature dependence and the risk of underestimating long-term migration by short-conditioning protocols [72]. Related storage studies reported MOSH migration after 9 months in the range 30–52 mg/kg for foods directly packed in paperboard, reinforcing that ”paper-based” does not inherently mean ”chemically inert”, especially in recycled systems [73]. This background matters for chemically modified cellulose because the modification chemistry may improve barrier performance but does not automatically eliminate the need to control legacy contaminants, adsorption–desorption effects, and recycling-loop accumulation [74].

In Europe, fiber-based materials for food contact often reference national guidance frameworks (e.g., Germany’s BfR Recommendation XXXVI for paper and board), which specify compositional restrictions and permitted use of additives for paper/board and molded fiber articles intended for food contact. While not harmonized EU-wide, such guidance documents frequently act as de facto benchmarks for market access and should be considered during the design of chemically modified cellulose intended for FCM applications [75].

Beyond the EU framework [1,2], key export markets impose additional substance-specific requirements for chemically modified cellulose: the US system relies on the FCN notification program under 21 CFR 176 managed by the FDA [4,7,68,69], while the Chinese system is based on the National Food Safety Standards, Japan (MHLW positive list introduced in 2020), and Southeast Asian markets increasingly require detailed declarations of conformity, including DS values and reagent specifications [75,76,77,78,79].

**Figure 4:**
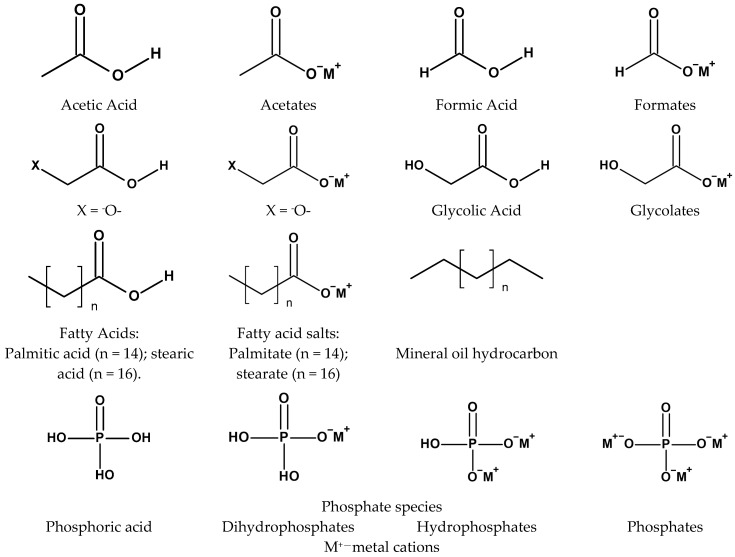
Representative low-molecular-weight motifs associated with non-intentionally added substances (NIASs) potentially relevant to chemically modified cellulose-based food contact materials: acetic acid/acetate species related to esterification and hydrolysis; fatty acids originating from long-chain ester modification; carboxymethyl-related fragments and counter-ion exchange products; small oxidation or aging-related fragments; phosphate-related species and associated counter-ions; and schematic mineral oil hydrocarbons representative of recycled-fiber contamination. The structures are illustrative and do not imply quantitative migration or toxicological relevance [2,43,73,76,77].

For chemically modified cellulose-based materials, NIAS concerns are most often associated with small, mobile species such as residual reactants, hydrolysis products, oxidation fragments, counter-ions, or legacy contaminants from recycled fibers rather than with the polymer backbone itself. Figure 4 illustrates representative low-molecular-weight chemical motifs discussed in this section to support the qualitative migration considerations summarized in Table 3 [2,43,73,76,77].

Beyond their influence on functional performance, chemical modification strategies applied to cellulose fibers may also affect the migration behavior of food contact materials by introducing new extractable species, modifying sorption characteristics, or altering aging and degradation pathways. These effects depend on the type and intensity of chemical modification, reaction localization, and the presence of residual reactants or by-products [2,43,77].

Table 3 summarizes migration and NIAS considerations for common cellulose modification strategies. Values represent literature trends, not regulatory limits [2,73,76].

### 5.2. Impact of Modification on Toxicological Profiles

Chemical modification can change food contact toxicology through three main routes: (1) altering the identity and quantity of migrants (residuals, by-products, and oligomers), (2) changing bioavailability and partitioning (e.g., increased hydrophobicity can shift migration into fatty foods), and (3) introducing reactive functionalities or inorganic/ionic components that behave differently during use and recycling. The safety profile is not a generic property of ”modified cellulose” but a function of chemistry class + process conditions + localization (surface vs. bulk) + use scenario [3,67].

A practical implication is that strategies often considered “low impact” from a materials perspective may still raise safety questions if reactive reagents or persistent ionic species are used. For example, cationization (e.g., quaternary ammonium functionalities) may reduce water uptake and tune surface charge, but it also creates a class of potential migrants (unreacted cationic reagents, counter-ions, and low-molar-mass quats) that require targeted analytical control and toxicological context. Similarly, phosphorylation can be favorable compared with fluorinated chemistries from a persistence standpoint, yet it introduces phosphorus-containing species whose leachability and behavior in recycling loops need to be quantified rather than assumed [3,51].

Because NIASs are frequently partially identified or present as mixtures, modern FCM safety discussions increasingly emphasize tiered risk assessment. The classic “threshold of regulation” framework (tiered approach) illustrates how exposure-driven thresholds can guide the need for toxicity testing for low-level migrants, provided that chemical identity/exposure can be bounded with defensible assumptions. In practice, this logic underpins why quantification (or at least bounding) of migration is essential even when full structural identification is incomplete [90].

At the same time, the scientific literature highlights structural gaps in conventional risk assessment for food contact materials: incomplete knowledge of chemical identities, insufficient hazard characterization for many migrants, and limited exposure data across realistic use patterns. These challenges are summarized in the widely cited Environmental Health Perspectives article on risk assessment of food contact materials, which calls attention to the mismatch between real-world chemical complexity and substance-by-substance regulatory evaluation. This framing is directly relevant for chemically modified cellulose, where the ”chemistry benefit” (barrier; wet strength) is often achieved by increasing chemical complexity at interfaces [91].

### 5.3. Safety-by-Design Approaches for Modified Cellulose

Safety-by-design for chemically modified cellulose starts with a simple operational principle: design the modification chemistry so that the plausible migrants are either (1) not hazardous, (2) not present, or (3) demonstrably non-migrating under intended conditions of use and recycling. Implementing this principle requires integrating chemistry selection, process control, and analytical verification from early development stages rather than treating safety as a late compliance exercise [3,91].

A practical NIAS-oriented framework is provided by best-practice guidance documents (e.g., ILSI Europe), which outline how to proceed when NIASs are present as mixtures and how to combine identification, exposure estimation, and hazard-based prioritization. For modified cellulose, this translates into: strict control of reagent purity and reaction quenching; explicit removal/neutralization of low-molar-mass residues; design preference for surface-selective, low-DS approaches that minimize bulk alteration and reduce the pool of extractables; and stress-testing under humidity/temperature cycling representative of packaging life [92].

Because chemical safety cannot be assessed on the basis of composition lists alone, targeted chemical analysis is increasingly complemented by bioassay-based screening (in vitro approaches) to capture mixture effects and unanticipated hazards, particularly in recycled or multilayer systems where unknown migrants may dominate. A comprehensive review of in vitro toxicity testing for food contact materials discusses feasibility, current assay families (cytotoxicity, genotoxicity, and endocrine activity), and FCM-specific challenges, offering a realistic pathway to strengthen safety evaluation where chemical identification is incomplete [93].

The food contact safety of chemically modified cellulose should not be treated solely as a downstream compliance step but rather as an inherent outcome of chemical design choices, processing conditions, and material architecture. Many strategies that reduce migration risk—such as low-DS, surface-selective modification; minimization of reactive residues; and controlled ionic interactions—also influence energy demand, chemical inputs, recyclability, and end-of-life behavior. As a result, safety-by-design and sustainability-by-design are tightly coupled rather than competing objectives in fiber-based packaging systems [2,3]. The following section, therefore, evaluates the chemical modification of cellulose through a life cycle perspective, examining when performance gains justify additional chemical complexity and how trade-offs between functionality, circularity, and environmental impact can be systematically assessed [93].

## 6. Sustainability Assessment of Chemically Modified Cellulose

### 6.1. Life Cycle Implications of Chemical Modification

Life cycle assessments of chemically modified cellulose separate environmental impacts into two domains. The chemical module includes reagents, solvents, neutralization, purification, and waste streams. The process module accounts for dewatering, drying, forming, coating, multilayer assembly, and logistics. This separation matters because many fiber modifications work at low DS with surface-localized chemistry. Here, wet-processing steps and drying operations typically dominate the environmental burden rather than the chemistry itself [94].

Nadeem et al. measured the cradle-to-gate impacts of CNF films (2 g; 100 g m^−2^): 0.241–0.426 MJ embodied energy and 0.018–0.034 kg CO_2_ eq GWP, depending on manufacturing route (spray deposition versus vacuum filtration). Normalizing by mass gives approximately 120–210 MJ kg^−1^ and 9–17 kg CO_2_ eq kg^−1^ for the film [95]. These values depend heavily on mechanical fibrillation intensity, dewatering efficiency, coating thickness (g m^−2^), and electricity mix. Process engineering choices, therefore, interact strongly with materials chemistry [95].

Chemical contributions increase when modifications require reagents with high upstream footprints, repeated washing, or generate saline or organic waste needing treatment. LCA studies of cellulose derivatives consistently show sensitivity to system boundaries, allocation rules, and inventory data. Laboratory-scale assessments extrapolate poorly to industrial implementation [94,96].

We, therefore, start by identifying the functional requirement and underlying chemical mechanism (grease or water vapor barrier, wet strength, and humidity resistance). Only then can we evaluate whether this mechanism is environmentally justified for the target packaging construction (basis weight, coating add-on, layer count, and end-of-life scenario). This logic favors surface-selective and low-add-on modifications that deliver functional gains without excessive chemical inputs or drying energy [94,95].

The EU Packaging and Packaging Waste Regulation (PPWR) entered force on 11 February 2025, with general application 18 months later. This regulatory context reinforces design strategies where chemical modification adds functionality without disrupting recycling streams or burdening existing infrastructure [97].

### 6.2. Recyclability and End-of-Life Compatibility

For fiber-based packaging, sustainability performance is determined less by bio-based origin than by behavior in conventional paper recycling, including repulping efficiency, separation of dispersed fractions, formation of stickies, fiber yield, pulp quality, and the ability to sustain multiple recycling cycles. CEPI guidelines emphasize that while paper is intrinsically recyclable, functional barriers—including coatings, laminates, and surface treatments—can interfere with recycling and, therefore, must be designed to remain compatible with industrial recycling processes under standard operating conditions [98].

In response, the European packaging sector is converging toward standardized technical assessment of recycling ability. The 4evergreen initiative has published the Fiber-Based Packaging Recyclability Evaluation Protocol (updated version 2025), which provides a harmonized framework for assessing technical recyclability across different process configurations, including conventional recycling mills, flotation-deinking scenarios, and specialized processes. Recycling modified cellulose fibers encounters a barrier of “hornification”. Drying irreversibly collapses the porous structure and creates strong internal hydrogen bonds. Each recycling cycle drastically reduces the fibers’ swelling capacity (WRV) and tensile strength (performance degradation). The literature data indicate that hornification is limited for cationic cellulose (low DS) compared to native cellulose, but the milling process during recycling causes rapid degradation of the polymer chain length. The protocol is based on experimental recycling data and enables comparative evaluation of packaging constructions with respect to real, process-relevant recyclability [98,99].

For cellulose modification chemistry, these developments translate into a concrete design criterion: whether a given chemical treatment behaves as a “disappearing” component during repulping or instead generates issues such as persistent hydrophobic coating fragments, increased sticky formation, deterioration of pulp brightness or color, or reduced fiber yield. Increasing emphasis is placed on modifications that are (a) hydrophilic or readily dispersible under repulping conditions, (b) applied at low add-on levels, and (c) as chemically homogeneous and behaviorally predictable as possible [98,99].

The 4evergreen Circularity by Design guidelines complement the Evaluation Protocol by providing expert-based recommendations on the compatibility of common barrier systems, coatings, and auxiliary components with recycling. These recommendations explicitly note that compatibility depends on both material class and chemistry and that constructions of uncertain behavior should be validated experimentally rather than assumed to be recyclable [100].

When end-of-life is considered beyond recycling alone (including energy recovery, landfilling, or composting), the critical question for paper-based constructions with barriers becomes whether the applied chemical modification is aligned with the intended EoL scenario, such as recycling in conventional paper mills. As a result, LCA and the ”design for recycling” literature increasingly treat recyclability as a boundary condition, with barrier performance optimized only, thereafter, to avoid a shift toward difficult-to-recycle composite structures [98,99,100].

### 6.3. Trade-Offs Between Performance and Circularity

The most challenging trade-offs in cellulose modification for food contact materials arise at the intersection of barrier performance (particularly against water vapor and grease), recycling ability, and chemical safety. Barrier layers that enhance moisture resistance often require fewer hydrophilic components or more continuous morphologies, which can impair disintegration during repulping and increase the risk of persistent residues. Conversely, barriers based exclusively on cellulose exhibit excellent oxygen barrier performance but remain highly sensitive to relative humidity, frequently necessitating additional architectural solutions such as multilayer moisture shielding [95,101].

LCA comparisons between “paper with barrier” and multilayer plastic films provide a useful benchmark for evaluating these trade-offs. In the study by Mousania et al. (Waste Management, 2025), which performed a cradle-to-grave comparison of multilayer plastic films with a paper-based alternative, the abstract reports 8.73–42.7% lower energy demand for coated paper-based packaging, depending on the material scenario [102]. At the same time, the study highlights that certain polymers (e.g., nylon-6) exhibit particularly high energy and climate burdens. While these findings support the general intuition of lower upstream energy demand for fiber-based solutions, they also underscore the necessity of critically evaluating system boundaries and end-of-life assumptions [102].

Reviews of biodegradable coatings further indicate that improvements in the water vapor barrier of paper are most commonly achieved using less polar biopolymers (e.g., PLA, PHB/PHBV) or hybrid systems. Although such approaches can be functionally effective, their overall sustainability performance depends strongly on coating mass, application process, and end-of-life scenario, particularly whether the resulting construction remains compatible with paper recycling or becomes a difficult-to-recycle composite [101].

From a chemistry-to-LCA perspective, the most promising design windows for fiber-based packaging are, therefore, those in which:

- Barrier functionality is achieved with minimal add-on (g/m^−2^) and/or selectively at the surface;
- The modification does not require intensive purification steps or generate chemically burdened waste streams;
- Recyclability is experimentally verified using industry-accepted protocols rather than merely declared [94,99,100].

Finally, it is increasingly recognized that in food packaging systems, food waste reduction can represent the dominant LCA “swing factor”. In some cases, a higher environmental footprint of the packaging material itself may be environmentally justified if it significantly extends shelf life and reduces product losses. Contemporary LCA reviews, therefore, treat this principle as essential for the correct interpretation of results, particularly when comparing packaging solutions with differing barrier functionalities [103].

## 7. Design Considerations for Modified Cellulose Packaging

### 7.1. From Chemical Modification to Quantifiable Performance Targets

A comparative design framework for chemically modified cellulose fibers must be grounded in quantifiable performance metrics, rather than in qualitative descriptors of chemical functionality. In fiber-based food packaging, the dominant failure modes—moisture sensitivity, grease penetration, humidity-induced loss of barrier, and wet strength decay—are controlled by interfacial phenomena that can be directly linked to the surface chemistry, degree of substitution (DS), and spatial localization of functional groups [95,98]. The choice of cellulose modification strategy for food contact is a compromise between chemical bond stability and physical barrier properties. The material’s adaptability depends on how the introduced functional groups react to food pH and extreme process temperatures. The chemical properties of food products determine the stability of modifications at the interface. Cellulose ethers (e.g., methylcellulose and ethylcellulose) demonstrate the best stability for acidic foods (pH < 4.5), as the ether bond is resistant to acid hydrolysis. However, modifications based on ester bonds (e.g., cellulose acetate) or cationization with amine groups can slowly degrade, leading to the migration of degradation products into the food and the loss of barrier properties, which poses a significant risk. In the case of packaging for fatty foods, increasing the surface energy of the fibers (hydrophobization) is crucial. Modifications using long-chain fatty acids are effective, as they create a dense crystalline network impermeable to lipids. However, it should be noted that traditional sizing agents (e.g., AKD) can dissolve in fats if the crosslinking process is not performed correctly [94,95,100,104,105,106].

The design strategy for cellulose food packaging should also take into account that thermal processes (such as high-temperature sterilization (>100 °C) or cooling and freezing) drastically affect the microstructure of modified fibers. High-temperature sterilization processes in the presence of steam cause fiber swelling and loosening of the nanocomposite structure. Thermal desorption of the modifying substances can occur. Therefore, it is advisable to use biological crosslinking agents with high thermal stability or covalent modifications with sufficiently stable linkages within controlled DS ranges, which maintain barrier integrity without compromising recyclability and food contact safety. In the cooling and freezing scenario, the main problem is not chemistry, but physics (water condensation and crystallization). Freeze–thaw cycles lead to microcracks in modified coatings (e.g., starch or alginate) due to the different thermal expansion of ice and cellulose. Adaptation to these processes can involve introducing plasticizers (e.g., glycerol) into the modified cellulose structure, which allows the coating to maintain flexibility at low temperatures and prevents flaking [94,95,100,104,105,106].

As a result, chemical modification strategies should be evaluated through standardized tests that translate molecular-scale changes into packaging-relevant performance indicators [98,104,105,106].

For moisture management, water absorptiveness measured by the Cobb method remains a critical metric, particularly for low-DS surface modifications and thin barrier layers. Even modest surface-localized chemical changes can reduce Cobb60 values substantially, despite minimal bulk substitution, highlighting the non-linear relationship between DS and functional outcome. In parallel, water vapor transmission rate (WVTR) testing (ASTM F1249) provides a standardized measure of humidity barrier performance across coated papers and multilayer fiber-based constructions, enabling comparison of surface-selective chemistry with thicker polymeric coatings. WVTR values are strongly dependent on relative humidity, underscoring that barrier performance cannot be interpreted independently of use conditions [104,106].

Grease resistance remains a particularly sensitive design target for fiber-based food contact materials. Operationally, it is still most often assessed using the Kit test (TAPPI T559), which reports resistance to increasingly aggressive oil mixtures up to a maximum rating of 12. While historically associated with fluorochemical treatments, the Kit test remains relevant as a comparative benchmark for PFAS-free alternatives. However, within a chemistry-driven framework, high Kit values must be interpreted alongside recyclability and safety considerations, as many grease-resistant chemistries rely on persistent hydrophobic domains that may compromise repulping or generate NIAS concerns [104].

For applications where oxygen sensitivity dominates product shelf life, oxygen transmission rate (OTR) measurements according to ASTM D3985 provide a common basis for comparing cellulose-based barrier layers, nanocellulose coatings, and polymeric references. Dense cellulose and nanocellulose networks can exhibit excellent oxygen barrier performance under dry conditions, but their sensitivity to humidity necessitates architectural solutions (e.g., multilayer designs) rather than increased chemical complexity alone [95,107].

Mechanical robustness, particularly for molded fiber products, represents another critical axis of the design framework. Chemical modification must be assessed in terms of dry versus wet strength retention, dimensional stability, and durability under cyclic moisture exposure. Industrially relevant data illustrate the magnitude of the challenge: dry tensile strengths on the order of 20–25 kN m^−1^ may drop to ~4–5 kN m^−1^ under wet conditions without appropriate wet-strength strategies. While covalent wet-strength systems can close this gap, they also introduce elevated risks for recycling ability and chemical safety, reinforcing the need to consider strength gains within a broader circularity context [108].

### 7.2. Integrating Circularity and Safety as Design Constraints

Recycling ability and food contact safety are hard constraints, not secondary optimization criteria. CEPI guidelines state that functional barriers, coatings, and surface treatments may interfere with conventional paper recycling and must remain compatible with industrial repulping, fiber recovery, and downstream processing [83]. The 4evergreen Fiber-Based Packaging Recycling Ability Evaluation Protocol defines harmonized testing routes for different recycling scenarios. Chemistry-generating persistent hydrophobic fragments, increasing sticky formation, and reducing fiber yield during repulping are high-risk regardless of laboratory barrier performance [99,100]. Surface-selective and low-add-on modifications minimize the disruption of fiber disintegration while enabling targeted performance gains. Bulk substitutions or thick hydrophobic layers may deliver robust barrier properties but often compromise recycling ability unless protocol testing demonstrates “disappearing” behavior during repulping [98,99,100].

Migration and NIAS risks (Section 5) increase with chemical complexity, reactive functionalities, and low-molecular-weight residues. Chemistries using minimal reagent inventories, stable covalent or ionic linkages, and low DS are inherently more robust from a safety-by-design perspective. More aggressive chemistries (e.g., covalent wet-strength systems) require stronger evidence packages: targeted migration testing and durability assessments under realistic use conditions [67,91].

In recent years, the emphasis on “green chemistry” has forced a shift away from toxic crosslinking agents (such as formaldehyde or epichlorohydrin) toward physical and biomimetic methods. These modern approaches are redefining the way we think about fiber durability and functionality. Plasma treatment (cold plasma) is a “dry” technique that uses ionized gas to activate the fiber surface without affecting its internal structure (bulk). The formation of free radicals on the cellulose surface facilitates subsequent grafting of polymers or directly increases hydrophobicity/hydrophilicity. The advantage of this method is the lack of water and chemical consumption and the extremely short reaction time (seconds/minutes). However, the disadvantage is the high investment cost in vacuum or atmospheric equipment; the modification effect can fade over time (so-called plasma aging). The industrial potential is high in the technical and medical textiles sector (sterilization + activation) [67,83,100].

Biological crosslinking agents allow for the replacement of toxic compounds with naturally derived substances. Genipin (derived from gardenia fruit) and nanoparticle-enhanced citric acid are attracting particular attention. Advantages include full biodegradability and zero toxicity (skin safety); high durability of the “non-iron” effect (anti-crease). Disadvantages include the very high cost of genipin (currently a barrier to mass production) and the tendency of fruit acids to yellow fibers at high temperatures [91,98,99].

Deep Eutectic Solvents (DESs) are a new class of green solvents (e.g., a mixture of choline chloride and urea) that are biodegradable and easily regenerated. An advantage is the ability to perform modifications (e.g., lignocellulose fractionation) at significantly lower temperatures than Kraft or sulfite processes. A disadvantage of this method is its high viscosity, which hinders mass transport in reactors; it is still in the optimization phase for large-scale recovery [67,99].

The greatest challenge for green technologies is scalability. While plasma processing is successfully entering the industry (roll-to-roll lines), enzymatic modifications or those based on expensive bio-crosslinkers are still economically unviable in mass production (e.g., in the production of CMC or cellulose acetate). The literature (e.g., green chemistry) emphasizes that the future belongs to hybrid processes—for example, short-duration plasma activation, which radically reduces the amount of chemicals required in the subsequent traditional step [83,98,100].

### 7.3. Defining Practical Design Windows

Synthesizing the relationships between chemistry, performance, safety, and circularity reveals a set of practical design windows for sustainable fiber-based food packaging. The most robust solutions typically occupy an intermediate regime in which functionality is achieved through surface-dominated interactions rather than extensive bulk modification. In this regime, modest chemical changes—often at DS values well below 0.1—can generate measurable improvements in water uptake, grease resistance, or wet stability while preserving repulp ability and minimizing chemical inventories [94,98].

Conversely, strategies that prioritize maximal barrier or strength performance through high substitution levels or continuous hydrophobic phases tend to shift the material into a high-risk domain, where recyclability, safety, and LCA performance become increasingly difficult to reconcile. Such approaches may still be justified for specific high-performance applications, but they require explicit acknowledgment of trade-offs and rigorous validation across the full life cycle [94,95,98,99,100].

The framework also accommodates the role of food waste reduction as a legitimate sustainability lever. LCA studies increasingly recognize that improvements in barrier performance that extend shelf life can offset higher material-level impacts, provided that such gains are real and quantifiable. Chemistry-driven design decisions should be evaluated not only against material-centric metrics but also against their potential to reduce product losses within the intended food system [103].

Overall, his comparative design framework positions chemical modification of cellulose as a precision tool, rather than a generic enhancement strategy. Explicitly linking chemical levers to measurable performance targets and constraining the solution space through recyclability and safety requirements provides a rational basis for translating laboratory-scale chemistry into industrially viable, sustainability-by-design, fiber-based food packaging systems.

Transitioning from the laboratory-based modification of cellulose fibers to industrial scale requires optimizing operating costs and adapting existing process lines. Key challenges include the scalability of chemical processes, which often require expensive or toxic solvents in the laboratory, which are difficult to recover on a large scale [98,100].

The primary goal of cost optimization is to reduce operating costs by increasing biomass yields and utilizing cheaper modification pathways. Chemical methods, such as alkalization (mercerization), are considered simple and economical compared to physical methods. Industry strives to utilize standard equipment, such as stirred batch reactors, pressure filtration systems, and spray dryers. Modifications such as esterification and etherification require precise temperature and pressure control (e.g., vacuum distillation to remove solvents like acetone). Ecology and recycling are key considerations, as is the pursuit of cost control by closing the reagent cycle. For example, in causticization processes, sodium hydroxide recovery can reach 95–98% [99,100,103].

The break-even point of a process depends on the type of modification and the target market. Building pilot-scale research stations (e.g., for pulp milling to obtain micro- and nanofibers) is a necessary step before industrial implementation. In mass production, modifications must be integrated into the continuous pulp production process. Profitability is primarily influenced by electricity prices and market demand.

The scale-up process typically involves three main phases:

- Laboratory phase (g): Reaction optimization, determination of DS and MS using instrumental methods (e.g., FTIR).
- Pilot phase (kg-t): Testing equipment adaptability, e.g., using composite grinding discs instead of basalt ones for improved nanostructure yield.
- Industrial phase (t/h): Full integration into the production line, where water removal (the most critical step in esterification) and automated process parameter control become crucial [98,99,100].

Table 4 below summarizes the key modification paths of cellulose fibers in terms of their process efficiency, costs and energy consumption.

## 8. Challenges and Future Directions

Bulk DS values can be misleading when substitution is spatially heterogeneous. Surface-enriched modification governs many functional properties more than uniform bulk chemistry. Low add-on treatments—surface-selective esterification, phosphorylation, or thin CNF/CNC coatings—produce measurable barrier improvements at DS levels that appear negligible when averaged across the entire fiber [10,38,109].

Characterization needs to reflect this spatial complexity. Beyond nominal DS, studies should report surface enrichment (XPS; ToF-SIMS), depth profiles (comparing XPS with ATR-FTIR), and stability under humidity cycling, varying ionic strength, and food simulant exposure. For industrial translation, we should report coating add-ons (g m^−2^), solid content, drying energy demand, and repulping behavior. Recyclability claims should be verified through harmonized test protocols rather than qualitative inference [98,99,110].

Humidity remains the dominant failure mechanism. Water sorption plasticizes hydrogen-bonded networks that provide oxygen barrier performance. Chemical functionalization alone rarely prevents this; architectural strategies (multilayer designs, hydrophobic shielding, or moisture-management topcoats) become necessary [101,111]. Single-point OTR and WVTR measurements can be misleading. Dynamic testing (OTR as a function of relative humidity and time following humidity transients) reflects real-world conditions more accurately. In one study, hot-pressing a 70 g m^−2^ CNF film reduced OTR from 516.7 to 3.6 cm^3^ m^−2^ day^−1^, with better retention at elevated humidity when latex shielding limited swelling at 90% RH [112]. TOCN coatings (~1.5 μm thick) reduce OTR at 0% RH but show much smaller benefits at 75% RH [111]. Humidity robustness is an architectural challenge, not simply a matter of increasing DS [107,111,112].

Bio-based sourcing does not guarantee chemical safety. Migration and NIAS profiles depend on specific reagents, residuals, side reactions, and aging pathways. Regulatory guidance recognizes NIAS formation from impurities, by-products, degradation, and storage interactions [88]. The FCM safety literature documents incomplete chemical inventories, limited hazard characterization, and mixture toxicity challenges. Problems intensify when reactive reagents, catalysts, or persistent hydrophobization chemistries are used [3,91].

EU overall migration limits (10 mg dm^−2^, commonly interpreted as 60 mg kg^−1^ under the 6 dm^2^/kg assumption) set the regulatory context for packaging materials. Standardized simulants, time/temperature protocols, and repeat-use scenarios become relevant for interpreting results [69,71]. New cellulose chemistries intended for food contact should include targeted and non-target screening of extractables and NIAS (LC-HRMS/GC-MS), migration testing under conservative conditions, characterization of aging-driven changes, and transparent reporting of reagent purity and purification efficiency [3,69,71,91,92].

Recyclability imposes operational boundaries. In European markets, functional barriers that interfere with repulping, fiber yield, or mill compatibility face practical and regulatory limits regardless of laboratory barrier data [98]. The 4evergreen Fiber-Based Packaging Recyclability Evaluation Protocol (updated April 2025) provides structured test methods for evaluating technical recyclability across different process configurations [99]. Studies reporting chemically modified cellulose should include protocol-relevant outcomes—disintegration behavior, residual fragments, stickies formation, and fiber yield impacts—particularly for hydrophobized layers, covalent wet-strength systems, and multilayer constructions [98,99,110].

Life cycle impacts often derive more from processing than from chemistry itself. Mechanical fibrillation, dewatering, and drying steps frequently dominate environmental footprints, especially when modifications add unit operations [96,113]. CNF-based layers show sensitivity to fibrillation intensity, deposition method, dewatering efficiency, and electricity source [111]. Meaningful LCA comparisons require transparent process inventories (water consumption, energy inputs, washing cycles, and solvent recovery assumptions) and sensitivity analyses across industrially plausible parameter ranges. Comparisons should maintain functional equivalence (barrier, mechanical properties, and shelf life) and account for food waste reduction as a legitimate packaging function [95,111,114,115].

Research priorities should emphasize surface-selective chemistries with low DS that can be characterized quantitatively and that retain stability under humidity cycling [10,38,109]. Humidity-resilient architectures remain essential, combining densification with protective topcoats or multilayer moisture barriers [111,112]. Recyclability evidence should come from protocol-based testing rather than inference [98,99,110]. Migration and extractables profiling, including NIASs, should be integrated into study designs from the outset [3,69,71,91,92]. Life cycle assessments need transparent process inventories and sensitivity analyses that reflect scale-up conditions [96,111].

Commercial deployment will require integrated demonstration studies that address performance, safety, and circularity under conditions representative of industrial processing and end-use environments.

## 9. Conclusions

Chemical modification can improve fiber packaging performance, but outcomes depend on how and where the chemistry is introduced. The practical benefit correlates more with substitution localization within the fiber wall than with the average degree of substitution. In multiple packaging-relevant studies, surface-enriched treatments at DS values below ~0.1 were sufficient to reduce moisture uptake and improve barrier response while maintaining repulp ability and recycling compatibility [80,116]. This exposes a reporting gap: bulk DS values often fail to capture the surface-dominated nature of heterogeneous reactions and can obscure spatial effects that determine functional performance.

Humidity sensitivity persists as a central limitation for cellulose- and nanocellulose-based oxygen barriers. Good OTR values under dry or moderate humidity conditions frequently deteriorate at elevated relative humidity because water sorption plasticizes the hydrogen-bonded network. Chemical functionalization alone rarely prevents this degradation. High-humidity robustness typically requires architectural solutions (multilayer constructions, hydrophobic shielding, or moisture-management topcoats) and should be evaluated through dynamic barrier testing rather than single-point measurements [95,112,117].

Food contact safety should be treated as a design consideration from the outset, not as a compliance check added later. Bio-based sourcing does not ensure low migration risk; extractables and NIAS profiles depend on reagent selection, residuals, side reactions, and aging under realistic conditions [3,91]. Low-DS surface modifications with stable linkages generally reduce the pool of potential migrants. More intensive chemistries require correspondingly stronger evidence packages, including targeted migration studies and aging tests.

Recyclability is a practical boundary condition for fiber-based packaging in European markets. Modifications that interfere with disintegration, fiber yield, or mill compatibility must be assessed through protocol-based recyclability testing rather than inferred from laboratory barrier measurements [98]. Recent life cycle assessments show that washing, dewatering, and drying steps often dominate environmental impacts [96,113], while an extended shelf life can offset higher packaging footprints when food waste reduction is accounted for [115]. The most transferable solutions combine surface-selective chemistry, limited process intensification, and verified circularity under realistic recycling conditions [3,80,91,95,96,98,112,113,115,116,117].

## Figures and Tables

**Table 1:** Overview of representative chemical modification strategies applied to cellulose fibers, typical ranges of degree of substitution (DS), and their primary functional implications relevant to fiber-based food packaging applications.

Modification Chemistry	Typical DS Range	Localization	Key Functional Effect	Packaging Relevance	Ref.
Acetylation	0.05–0.30	surface/ near-surface	↓* moisture uptake, ↓ WVTR	moisture barrier	[40,54,55]
Fatty acid esterification	0.02–0.15	surface-dominant	↑ KIT grease resistance (KIT 7–10)	grease barrier	[56,57]
Etherification (CMC)	0.05–0.20	bulk/surface	↑ bonding, ↑ swelling	strength, formation	[58]
Phosphorylation (P(V))	<0.10	surface-selective	ionic crosslinking, ↑ wet stability	molded fiber	[39,59,60]
TEMPO oxidation	0.05–0.15	surface	↑ bonded area, ↑ O_2_ barrier (dry)	gas-barrier layers	[33,61]

↓*—decrease; ↑—increase.

**Table 2:** Representative barrier ranges of chemically modified cellulose-based systems, with CNF coating included as a reference benchmark, for fiber-based food packaging. Reported ranges reflect commonly observed literature values rather than best-case records; values depend strongly on relative humidity (RH), coating add-on, and substrate structure.

Modification Strategy/Nanocellulose Systems	OTR (23 °C, 50% RH) (cm^3^·m^−2^·day^−1^·bar^−1^)	WVTR (23 °C, 50% RH) (g·m^−2^·day^−1^)	Grease Resistance (KIT)	RH Sensitivity	Ref.
Unmodified paper/board	>>1000	~800–1200	0–1	very high	[5,21,22]
CNF coating (dry or low RH)	<10	>500	1–2	extreme	[28,61,65]
Acetylated fibers (low DS)	50–300	300–600	2–4	moderate	[41,55]
Fatty-acid-modified fibers	200–600	150–400	7–10	low-moderate	[56,57]

**Notes:** OTR—oxygen transmission rate; WVTR—water vapor transmission rate; RH—relative humidity; DS—degree of substitution; CNF—cellulose nanofibril; KIT—KIT grease resistance value (according to TAPPI T559); low DS—low degree of substitution. CNF coatings are included as reference benchmarks. Multilayer constructions combining nanocellulose with hydrophobic shielding layers fall outside the substrate-level chemical modification scope of this review.

**Table 3:** Representative migration- and NIAS-related considerations associated with common chemical modification strategies for cellulose fibers used in food contact materials.

Cellulose Modification Strategies	Typical Source of Potential Migrants/NIAS	Qualitative Migration Concern	Ref.
**Acetylation** **(low DS)**	Residual acetic species; low-molecular-weight esters; hydrolysis products	Generally low at controlled DS; increases with aging	[78,79,80]
**Long-chain fatty-acid esterification**	Fatty acid fragments; residual catalysts or solvents	Relevant mainly for fatty foods and long contact times	[81,82]
**Etherification (CMC-type)**	Unreacted etherifying agents; counter-ions; oligomeric residues	Elevated if grafted; lower when used as additive	[83,84]
**TEMPO-mediated oxidation**	Oxidation by-products; residual salts	Moderate; sensitive to humidity and aging	[85,86]
**Phosphorylation (P(V))**	Phosphate species; counter-ion exchange products	Generally moderate; chemistry- and ion-dependent	[73,87,88]
**Multivalent-ion complexation**	Leachable metal ions (Ca^2+^, Mg^2+^, Al^3+^)	Low when food-approved ions are used	[77]
**Recycled fibre background**	MOSH/MOAH; printing-ink-related NIASs	High unless functional barriers are applied	[76,89]

**Notes:** NIAS—non-intentionally added substance; DS—degree of substitution; CMC—carboxymethyl cellulose; TEMPO—2,2,6,6-tetramethylpiperidine-1-oxyl (mediated oxidation); P(V)—pentavalent phosphorus-based phosphate chemistry; MOSH—mineral oil saturated hydrocarbon; MOAH—mineral oil aromatic hydrocarbon.

**Table 4:** Key paths for modifying cellulose fibers in terms of their process efficiency, costs and energy consumption.

Cellulose Modification Strategies	Reaction Efficiency	Relative Cost	Energy Consumption	Process Characteristics	Ref.
Etherification (e.g., CMC)	High (70–90%)	Medium	Low/Medium	Water–alcohol processes; easy solvent regeneration	[40,54,55,78,79,80]
Esterification (e.g., CA)	Medium (50–75%)	High	High	Requires anhydrides and acids; energy-intensive distillation and acid recovery	[56,57,81,82]
TEMPO oxidation	High (up to 100%)	Very High	Medium High	High catalyst cost; precise pH control required	[33,61,83,84,85,86]
Enzymatic modification	Very High	High (enzymes)	Very Low	Green process but limited to low DS values	[83,85,98,100]

## Data Availability

No new data were created or analyzed in this study. Data sharing is not applicable to this article.

## Abbreviations

The following abbreviations are used in this manuscript:

AGU	Anhydro glucose unit
ATR-FTIR	Attenuated total reflectance Fourier-transform infrared spectroscopy
BfR	Bundesinstitut für Risikobewertung (German Federal Institute for Risk Assessment)
Ca^2+^	Calcium ion
CEPI	Confederation of European Paper Industries
CMC	Carboxymethyl cellulose
CNFs	Cellulose nanofibrils
CNCs	Cellulose nanocrystals
Cobb60	Water absorptiveness measured over 60 s (ISO 535)
DS	Degree of substitution
EoL	End of life
EU	European Union
FCM	Food contact material
FCC	Food contact chemical
GWP	Global warming potential
HEC	Hydroxyethyl cellulose
ISO	International Organization for Standardization
KIT	Grease resistance rating (TAPPI T559)
LCA	Life cycle assessment
MOAHs	Mineral oil aromatic hydrocarbons
MOSHs	Mineral oil saturated hydrocarbons
NIAS	Non-intentionally added substances
OML	Overall migration limit
OTR	Oxygen transmission rate
PFAS	Per- and polyfluoroalkyl substances
PLA	Polylactide
PPWR	Packaging and Packaging Waste Regulation
P(V)	Pentavalent phosphorus
RH	Relative humidity
SML	Specific migration limit
TEMPO	2,2,6,6-tetramethylpiperidine-1-oxyl
TOCNFs	TEMPO-oxidized cellulose nanofibrils
WVTR	Water vapor transmission rate
XPS	X-ray photoelectron spectroscopy
